# Equine sentinels and one health: a comprehensive serological survey of Crimean-Congo hemorrhagic fever virus in southeastern and Central Europe

**DOI:** 10.3389/fvets.2026.1810641

**Published:** 2026-04-24

**Authors:** Jignesh Italiya, Pavlina Nekudova, Petr Vaclavek, Nikolina V. Rusenova, Anton G. Rusenov, Andrei D. Mihalca, Florica Bărbuceanu, Vlad B. Vuta, Doru V. Hristescu, David Modry, Lisa E. Hensley, Anne W. Rimoin, Ludek Zurek

**Affiliations:** 1 Department of Microbiology, Nutrition and Dietetics, Czech University of Life Sciences, Prague, Czechia; 2 Department of Virology, State Veterinary Institute, Jihlava, Czechia; 3 Department of Veterinary Microbiology, Infectious and Parasitic Diseases, Trakia University, Stara Zagora, Bulgaria; 4 Department of Internal Diseases, Trakia University, Stara Zagora, Bulgaria; 5 Department of Parasitology and Parasitic Diseases, University of Agricultural Sciences and Veterinary Medicine of Cluj-Napoca, Cluj-Napoca, Romania; 6 Parasitology Consultancy Group, Corușu, Romania; 7 Institute for Diagnosis and Animal Health, Bucharest, Romania; 8 Faculty of Veterinary Medicine, University of Agricultural Sciences and Veterinary Medicine of Bucharest, Bucharest, Romania; 9 Department of Veterinary Disciplines, Czech University of Life Sciences, Prague, Czechia; 10 Department of Botany and Zoology, Faculty of Science, Masaryk University, Brno, Czechia; 11 Institute of Parasitology, Biology Center of Czech Academy of Sciences, České Budějovice, Czechia; 12 Zoonotic Emerging Disease Research Unit, US Department of Agriculture, Manhattan, KS, United States; 13 Department of Epidemiology, UCLA Fielding School of Public Health, Los Angeles, CA, United States

**Keywords:** Bulgaria, CCHFV, Czech Republic, domestic equine, Romania, serological surveillance

## Abstract

Crimean-Congo hemorrhagic fever (CCHF) is the widespread tick-borne viral disease affecting humans with endemic circulation across Africa, Asia, Eastern and Southern Europe. Climate change, global travel, and animal trade have accelerated the expansion of *Hyalomma* spp., the primary tick vectors of CCHF, into previously non-endemic areas, including the Czech Republic. To assess the current distribution of CCHF in equids across Southeastern and Central Europe, we screened serum samples from horses and donkeys from Bulgaria (*n* = 579), Romania (*n* = 1,534), and the Czech Republic (*n* = 576) by the ELISA kit to detect anti-CCHF antibodies. We observed a clear southeast-to-northwest gradient in seroprevalence: 14.5% (Bulgaria), 1.5% (Romania), and 0% (Czech Republic). These findings correlate with regional environmental conditions and vector distribution and indicate the northward spread of *Hyalomma* spp. This suggests that climate- and land-use driven ecological changes create conditions for CCHF circulation in regions previously considered low risk.

## Introduction

Crimean-Congo hemorrhagic fever virus (CCHFV) is a zoonotic, tick-borne virus belonging to the family *Nairoviridae,* genus *Orthonairovirus*. It can cause severe hemorrhagic fever in humans, with case fatality rates ranging from 10 to 40% (1). CCHF is primarily transmitted through the bite of *Hyalomma* ticks. The geographic distribution of the virus closely mirrors that of its primary tick vectors, and the enzootic cycle involves ticks and domestic and wild mammals (2). While CCHF has been traditionally endemic to parts of Africa, the Middle East, Asia, and Southeastern Europe, the tick vectors have begun to expand their range northward (3). This expansion is attributed to factors such as global warming, shifts in land use, altered migratory bird patterns, and increasing animal and human mobility, collectively driving the establishment of competent vectors in new regions. Consequently, this poses a new and growing public health concern in Central and Western Europe (4, 5).

*Hyalomma* ticks are recognized as prevalent ectoparasites and disease vectors impacting a wide array of livestock. Historically, domestic equines have been used as hosts for monitoring arboviruses due to their proximity to human populations and frequent exposure to arthropod vectors (6, 7). The likelihood of detecting *Hyalomma* ticks is higher in horses, as they are routinely groomed and closely monitored, and the ticks are easily recognizable to the genus level based on their morphology, even from photographs submitted through citizen science projects. This is supported by numerous *Hyalomma* tick reports on horses in Central, Western, and Northern Europe, including the Czech Republic, Austria, Hungary, Poland, the Netherlands, the United Kingdom, Sweden, and Norway (8–15). While the main mode of transportation of ticks is not known, it is assumed to be via the immature stages carried by migratory birds (8–15).

Despite increasing concerns over CCHFV spread in Europe, sero-epidemiological data on virus exposure of domestic equids remain limited. To date, no coordinated multi-country equine serosurvey has been conducted across Southeaster and Central Europe, representing a major gap in early warning surveillance. The primary objective of this study was to determine the seroprevalence of anti-CCHFV antibodies and to evaluate the potential CCHFV expansion from Southeastern into Central Europe using domestic equines as sentinel animals.

## Materials and methods

All samples were obtained by authorized veterinarians as part of national surveillance efforts for other research or pathogens surveillance. To ensure the study was sufficiently powered to detect low-level seroprevalence, a minimum sample size of n ≈ 385 per country was targeted using the standard epidemiological formula: *n* = *Z*^2^*P*(1 − *P*)/*d*^2^, where *Z* is the *Z*-score for the desired confidence level (1.96 for 95%), P is the expected prevalence (assumed 50%), and d is the desired precision (5%). This calculation was performed using a 95% confidence level and 5% precision based on a 50% expected prevalence (the most conservative estimate when true prevalence is unknown) (16, 17). Thus, we sampled more extensively horses and donkeys in Romania where CCHFV prevalence is unknown, but it is expected to potentially expand there from Bulgaria. Samples were obtained with prioritizing geographic diversity and regions with known *Hyalomma* tick expansion to ensure sufficient power for detecting low-level seroprevalence.

Overall, 2,689 serum samples of domestic equines (horses and donkeys) were used to detect the distribution of CCHFV across Southeastern and Central Europe. The samples included 576 horse sera collected in 2024 across the Czech Republic; 1,534 horse sera from 15 counties in Romania collected in 2025; and 579 horse and donkey sera collected from 17 provinces in Bulgaria in 2015 (200 donkeys) and 2024 (65 donkeys and 314 horses) (Supplementary Tables S1–S3). Sera were kept at −80 °C before use. Serological analysis was conducted using the commercial ID Screen^®^ CCHF Double Antigen Multi-species ELISA kit (IDvet, France), following the manufacturer instructions. This kit utilizes a double-antigen sandwich ELISA (DAS) principle where microwells are pre-coated with purified recombinant CCHFV nucleoprotein (NP). Specific antibodies in the sample are captured by the immobilized NP and subsequently detected by a horseradish peroxidase (HRP)-labeled recombinant NP conjugate. This “sandwich” mechanism enables the detection of total anti-CCHFV antibodies without the need for species-specific secondary antibodies. ELISA results were quality-controlled using internal kit standards. Descriptive statistics (95% Confidence Intervals using the Wilson score method for binomial proportions and Chi-square test in Microsoft Excel) were used to calculate seroprevalence in each region and species group.

## Results and discussion

**Figure 1:**
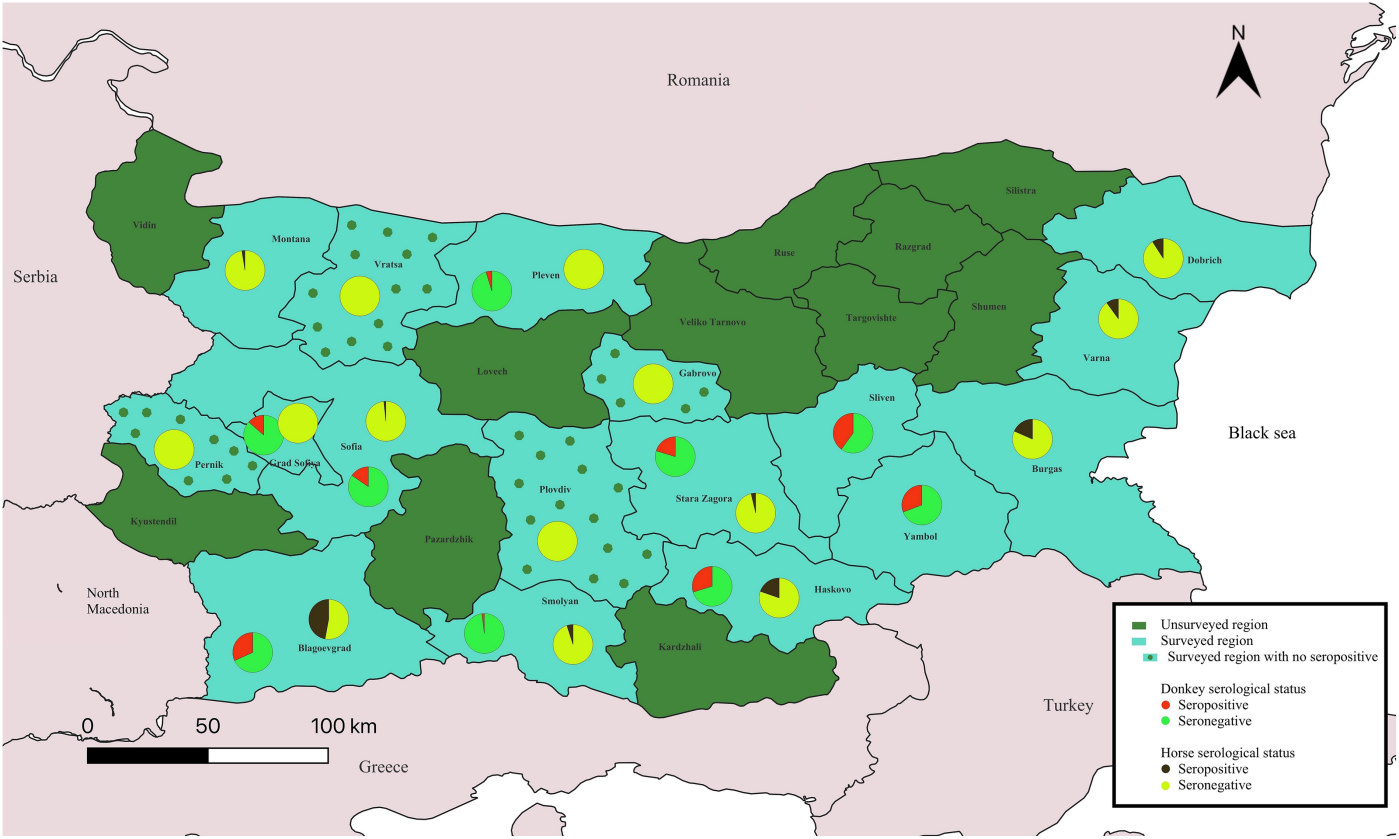
Geographic distribution and seroprevalence of Crimean-Congo hemorrhagic fever virus (CCHFV) in equids in Bulgaria. Pie charts display the proportion of seropositivity and seronegative equids in each province. Detailed sample information is provided in Supplementary Tables S1.1, S1.2.

In Bulgaria, anti-CCHFV-specific antibodies were detected in donkeys and horses with an overall seroprevalence of 14.5% (84/579; 95% confidence interval [CI]: 11.8–17.6%). Interestingly, donkeys showed a significantly higher seroprevalence of 18.9% (50/265; 95% CI: 14.6–23.9%) compared to 10.8% in horses (34/314; 95% CI: 7.8–14.8%) (Chi-square test, *χ*^2^ = 7.3, *p* = 0.007). These results demonstrate extensive CCHFV exposure across 17 Bulgarian provinces, providing compelling evidence of widespread viral activity including northern and eastern provinces bordering Romania, Serbia, and North Macedonia (Figure 1). These findings are in agreement with previous nationwide surveillance reports indicating CCHFV circulation in Bulgarian livestock (18–20). The detected prevalence rates in equids reinforce their utility as sentinel species capable of signaling local virus activity. Bulgaria is a CCHFV endemic country, where human cases are reported annually with most incidences concentrated the south-central provinces such as Kardzhali and Haskovo, as well as in southeastern Burgas (21). This is in agreement with our survey, where we detected positive horses or donkeys in provinces Haskovo and Burgas (no samples were analyzed from Kardzali).

**Figure 2:**
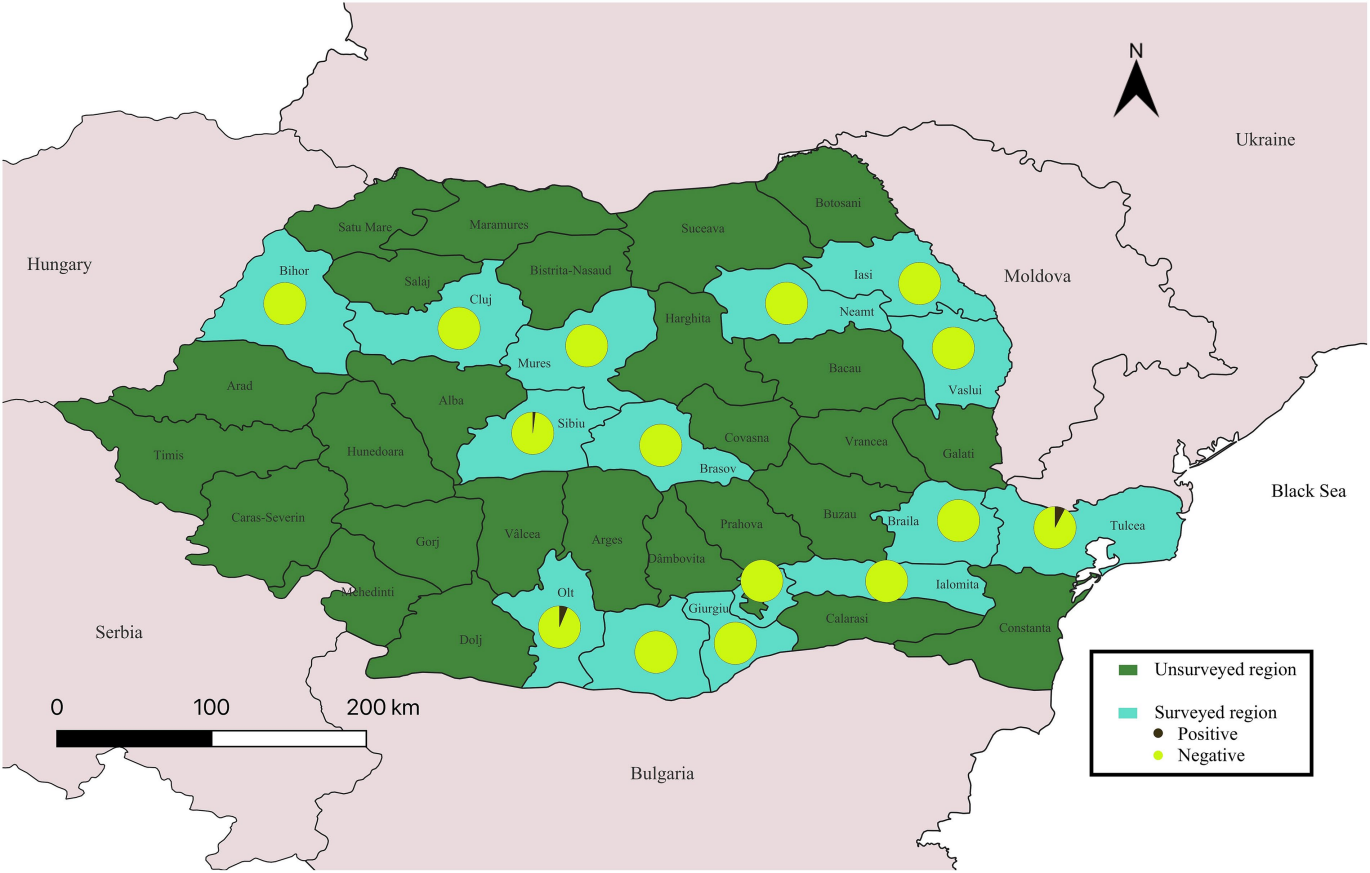
Geographic distribution and seroprevalence of Crimean-Congo hemorrhagic fever virus (CCHFV) in equids across Romania. Pie charts illustrate the proportion of seropositive and seronegative detected in each surveyed county. Detailed sample information is provided in Supplementary Table S2.

In Romania, 23 out of 1,534 equine serum samples tested seropositive for CCHFV, corresponding to an overall seroprevalence of 1.5% (23/1534; Figure 2) (95% CI: 1.0–2.2%). The highest seroprevalence was detected in Tulcea County, an area characterized by steppe vegetation, extensive livestock grazing, and where CCHFV seroprevalence in small ruminants has previously been reported (22–24), suggesting potential localized endemic foci of viral circulation. A single positive sample was also detected in Olt County, which shares a border with Bulgaria Pleven district, where positive equids are reported in this study as well. Furthermore, two positive horses were among 100 samples analyzed from the Sibiu County. According to the most recent reports on *Hyalomma* spp. distribution, established populations of this tick species are found primarily in southern Romania, bordering Bulgaria and Serbia as well as along the southeastern border with Hungary (3). *Hyalomma* spp. have not previously been documented from the Sibiu County (25). This unexpected detection in a non-endemic area warrants further investigation, as it may represent introduction via animal movement, sporadic introduction of infected ticks via migratory birds, or climate-related changes enabling tick expansion and establishment.

**Figure 3:**
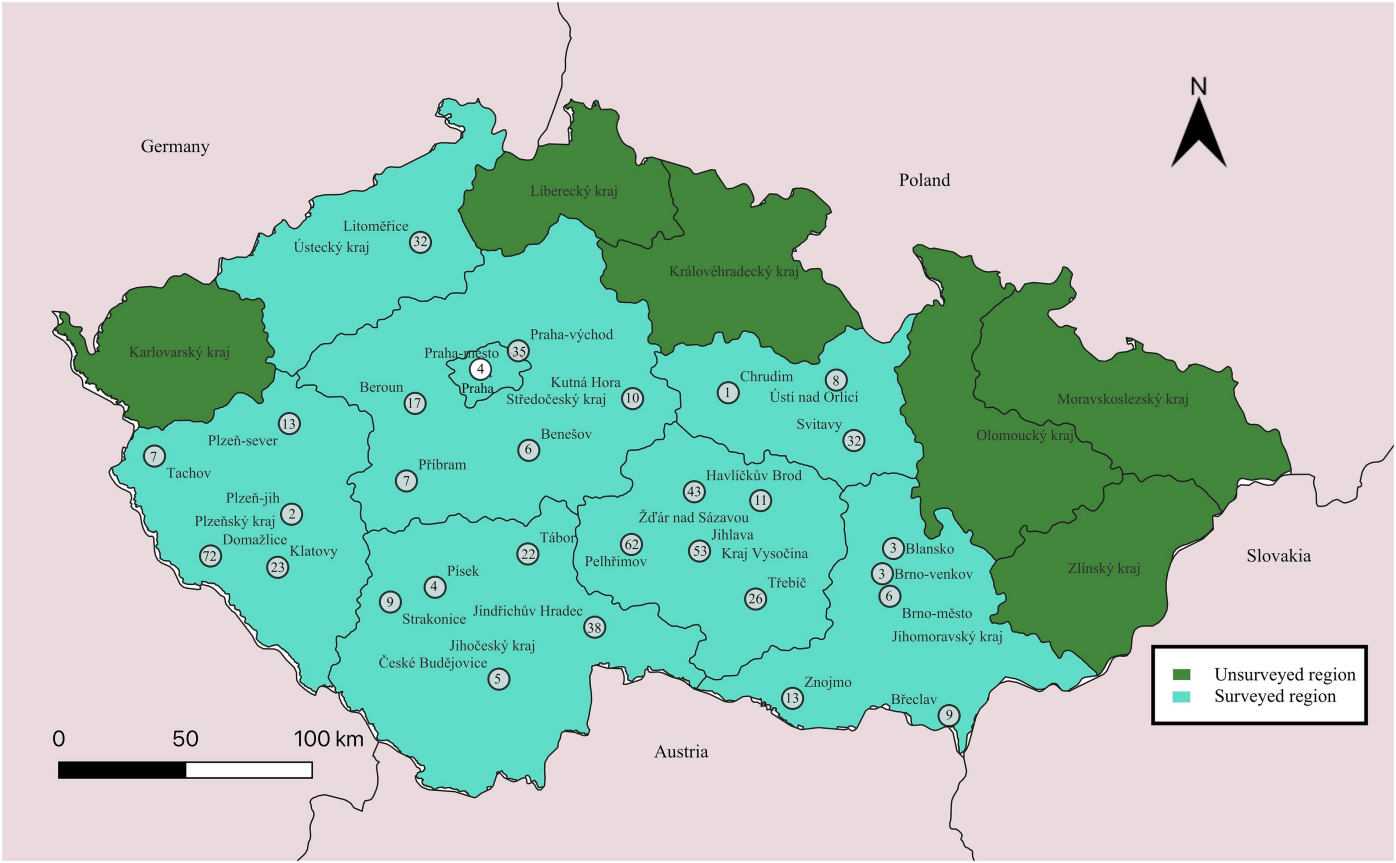
Geographic distribution and seroprevalence of Crimean-Congo hemorrhagic fever virus (CCHFV) in equids across the Czech Republic. Numbers within each circle indicate the number of samples screened at the corresponding location. Detailed sample information is provided in Supplementary Table S3.

In the Czech Republic, serological screening of 576 horses demonstrated an absence of detectable anti-CCHFV antibodies (0%; 95% CI: 0.0–0.6%). The sero-surveillance focused on regions where occasional findings of individuals of *Hyalomma* spp. had previously been reported on equids (8), specifically the Středočeský, Plzeňský, Jihomoravský, and Ústecký regions as well as other parts of the country (Figure 3).

Overall, our data demonstrate a geographical gradient of CCHFV exposure, characterized by elevated seroprevalence in Bulgaria and emerging focal detection in southern Romania. The absence of antibodies in the Czech Republic demonstrates that stable viral cycles are not established in this part of Central Europe yet. These results emphasize the need of cross-border equid surveillance to monitor the potential northward expansion of CCHFV.

In the natural cycle of CCHFV, virus transmission occurs between vectors and wild and domestic animals. In Bulgaria, main tick vectors of CCHFV, *Hyalomma marginatum* and *H. aegyptium,* are known established species (3). In Romania, *H. marginatum* is considered established primarily in southern and southeastern regions, where it has been collected from diverse hosts including livestock, birds, and lizards; additionally, other species such as *H. aegyptium* and *H. detritum scupense* have also been observed (26). In the Czech Republic, it has been shown that *H. marginatum* and *H. rufipes* are introduced via migratory birds and have been detected on horses (8, 27, 28). It has been suggested that the changing environmental conditions in Central Europe are conducive for establishment of permanent populations in this region, but this has not been fully demonstrated yet (8, 27, 28).

In wild and domestic animals, CCHFV exposure results in short-lived viremia, e.g., 2–3 days in ruminants (29). Although in equids experimental infections demonstrated no clinical signs or virus amplification, consistent antibody responses have been well described (30, 31).

Our study utilized a commercially available ELISA kit for anti-CCHFV antibody detection, which exhibits a specificity of 100% and a sensitivity of 98.9% across multiple species. Independent evaluations have similarly confirmed high performance, with specificity of 100% and sensitivity of 99.9%, although the possibility of false-positive or false-negative results cannot be fully excluded (32). Such outcomes may arise from cross-reactivity with antibodies against related viruses or from low antibody titters in early or late stages of infection. In future studies, confirmatory tests such as virus neutralization tests (VNT) need to be performed to confirm CCHFV-specific antibodies. It is also important to point out that this assay cannot determine the precise time of infection since antibodies may persist for months after virus exposure.

Overall, our findings indicate that examined Czech horses were not exposed to CCHFV even though the tick vector has been reported in the country (8). In contrast, equids from Bulgaria frequently carried antibodies, and some Romanian regions also showed evidence of exposure, including areas where the primary vector has not been reported. Equids therefore serve as valuable sentinel animals for CCHFV surveillance because they are closely monitored by their owners as part of routine care and develop detectable CCHFV antibodies following exposure, providing evidence of previous virus exposure. However, their utility is limited as indicators of active infection because equids do not develop significant viremia and are incapable of amplifying the virus.

This investigation represents the largest multi-country CCHFV serosurveys in equids in the region and offers critical baseline data for anticipating future spread of CCHFV in non-endemic areas. The changing dynamics in Europe mirror similar ecological shifts seen elsewhere, including parts of North America, underscoring the importance of global CCHF surveillance and preparedness. As vector ranges expand, the U. S. and other non-endemic countries must evaluate their own animal and human exposure risks and invest in proactive, One Health-driven strategies for detection, prevention, and response. Climate change, environmental degradation, and globalization have resulted in the proliferation of numerous vectors and the diminishment of transmission barriers, heightening the danger of vector-borne diseases in areas formerly deemed low-risk (33). The introduction and establishment of *Hyalomma* ticks into non-endemic parts of Europe, likely facilitated by migratory birds and climate-induced habitat changes, demonstrates the need for robust surveillance systems and integrated response strategies (3). The current investigation can serve as a model for non-endemic regions to proactively address emerging vector-borne threats using equine sentinels within a One Health surveillance system.

## Data Availability

The original contributions presented in the study are included in the article/Supplementary material, further inquiries can be directed to the corresponding author.

## References

[1] World Health Organization. Crimean-Congo haemorrhagic fever. (2023). Available online at: https://www.who.int/news-room/fact-sheets/detail/crimean-congo-haemorrhagic-fever (Accessed August 1, 2025).

[2] Zivcec M Scholte FEM Spiropoulou CF Spengler JR Bergeron É. Molecular insights into Crimean-Congo hemorrhagic fever virus. Viruses. (2016) 8:106. doi: 10.3390/v8040106, 27110812 PMC4848600

[3] Celina SS Černý J. *Hyalomma marginatum* in Europe: the past, current status, and future challenges—a systematic review. Transbound Emerg Dis. (2025) 2025:7771431. doi: 10.1155/tbed/7771431, 40765715 PMC12324920

[4] Alves Rodrigues M Lesiczka P Fontes MC Cardoso L Coelho AC. The expanding threat of Crimean-Congo haemorrhagic fever virus: role of migratory birds and climate change as drivers of *Hyalomma* spp. dispersal in Europe. Birds. (2025) 6:31. doi: 10.3390/birds6020031

[5] Dom C Sándor AD Mihalca AD. Climate change and species distribution: possible scenarios for thermophilic ticks in Romania. Geospat Health. (2016) 11:421. doi: 10.4081/gh.2016.421, 27245802

[6] Arshad A Reif AH Cavalleri JMV Desvars-Larrive A. Zoonotic pathogens in equids in Central Europe: a systematic review. BMC Vet Res. (2025) 21:451. doi: 10.1186/s12917-025-04915-5, 40629389 PMC12235778

[7] García-Bocanegra I Arenas-Montes A Jaén-Téllez JA Napp S Fernández-Morente M Arenas A. Use of sentinel serosurveillance of mules and donkeys in the monitoring of West Nile virus infection. Vet J. (2012) 194:262–4. doi: 10.1016/j.tvjl.2012.04.017, 22633828

[8] Lesiczka PM Daněk O Modrý D Hrazdilová K Votýpka J Zurek L. A new report of adult *Hyalomma marginatum* and *Hyalomma rufipes* in the Czech Republic. Ticks Tick Borne Dis. (2022) 13:101894. doi: 10.1016/j.ttbdis.2021.101894, 34996002

[9] Duscher GG Kienberger S Haslinger K Holzer B Zimpernik I Fuchs R . Hyalomma spp. in Austria—the tick, the climate, the diseases and the risk for humans and animals. Microorganisms. (2021) 10:1761. doi: 10.3390/pathogens10091761 PMC9502680 36144363

[10] Földvári G Szabó É Tóth GE Lanszki Z Zana B Varga Z . Emergence of *Hyalomma marginatum* and *Hyalomma rufipes* adults revealed by citizen science tick monitoring in Hungary. Transbound Emerg Dis. (2022) 69:e2240–8. doi: 10.1111/tbed.14563, 35436033 PMC9790508

[11] Nowak-Chmura M Siuda K Wegner Z Piksa K. Species diversity of ticks (Acari: Ixodida) on migrating birds on the Baltic Sea coast of Poland. Zool Stud. (2012) 51:1056–63.

[12] Uiterwijk M Ibáñez-Justicia A van de Vossenberg B Jacobs F Overgaauw P Nijsse R . Imported Hyalomma ticks in the Netherlands. Parasit Vectors. (2021) 14:244. doi: 10.1186/s13071-021-04738-x 33962655 PMC8106226

[13] Hansford KM Carter D Gillingham EL Hernandez-Triana LM Chamberlain J Cull B . *Hyalomma rufipes* on an untraveled horse: first evidence of Hyalomma nymphs moulting in the United Kingdom. Ticks Tick Borne Dis. (2019) 10:704–8. doi: 10.1016/j.ttbdis.2019.03.003 30876825

[14] Grandi G Chitimia-Dobler L Choklikitumnuey P Strube C Springer A Albihn A . First records of adult *Hyalomma marginatum* and *H. rufipes* ticks in Sweden. Ticks Tick Borne Dis. (2020) 11:101403. doi: 10.1016/j.ttbdis.2020.101403 32037097

[15] Hasle G Bjune G Edvardsen E Jakobsen C Linnehol B Røer JE . Transport of ticks by migratory passerine birds to Norway. J Parasitol. (2009) 95:1342–51. doi: 10.1645/GE-2146.1, 19658452

[16] Stevenson MA. Sample size estimation in veterinary epidemiologic research. Front Vet Sci. (2021) 7:539573. doi: 10.3389/fvets.2021.539573, 33681313 PMC7925405

[17] Naing L Nordin RB Abdul Rahman H Naing YT. Sample size calculation for prevalence studies using Scalex and ScalaR calculators. BMC Med Res Methodol. (2022) 22:209. doi: 10.1186/s12874-022-01681-1 35907796 PMC9338613

[18] Christova I Panayotova E Groschup MH Trifonova I Tchakarova S Sas MA. High seroprevalence for Crimean-Congo haemorrhagic fever virus in ruminants in the absence of reported human cases in many regions of Bulgaria. Exp Appl Acarol. (2018) 75:227–34. doi: 10.1007/s10493-018-0258-7, 29713918

[19] Mertens M Schuster I Sas MA Vatansever Z Hubalek Z Güven E . Crimean-Congo hemorrhagic fever virus in Bulgaria and Turkey. Vector Borne Zoonotic Dis. (2016) 16:619–23. doi: 10.1089/vbz.2016.1944, 27467142

[20] Barthel R Mohareb E Younan R Gladnishka T Kalvatchev N Moemen A . Seroprevalence of Crimean-Congo haemorrhagic fever in Bulgarian livestock. Biotechnol Biotechnol Equip. (2014) 28:540–2. doi: 10.1080/13102818.2014.931685, 26019541 PMC4434116

[21] Ngoc K Stoikov I Trifonova I Panayotova E Taseva E Trifonova I . Molecular and clinical characterization of Crimean-Congo hemorrhagic fever in Bulgaria, 2015–2024. Pathogens. (2025) 14:785. doi: 10.3390/pathogens14080785, 40872295 PMC12389410

[22] Bratuleanu B Anita A Temmam S Dascalu A Crivei L Cozma A . Seroprevalence of Crimean-Congo hemorrhagic fever among small ruminants from southern Romania. Vector Borne Zoonotic Dis. (2022) 22:397–401. doi: 10.1089/vbz.2021.0091, 35772004

[23] Ceianu CS Panculescu-Gatej RI Coudrier D Bouloy M. First serologic evidence for the circulation of Crimean-Congo hemorrhagic fever virus in Romania. Vector Borne Zoonotic Dis. (2012) 12:718–21. doi: 10.1089/vbz.2011.0768, 22897346

[24] Raileanu C Anita A Porea D Savuta G. Serologic evidence of Crimean-Congo haemorrhagic fever infection in small ruminants in southeastern Romania. Epidemiol Sante Anim. (2015) 67:145–9.

[25] European Centre for Disease Prevention and Control. The spatial distribution of Crimean-Congo haemorrhagic fever in Europe and its neighbours. (2023). Available online at: https://www.ecdc.europa.eu/sites/default/files/documents/crimean-congo-haemorrhagic-fever-spatial-distribution-december-2023.pdf (Accessed August 1, 2025).

[26] Coipan EC Vladimirescu AF Ciolpan O Teodorescu I. Tick species (Acari: Ixodoidea) distribution, seasonality and host associations in Romania. Travaux du Muséum National d'Histoire Naturelle "Grigore Antipa". (2011) 54:301–17. doi: 10.2478/v10191-011-0018-y

[27] Hubálek Z Sedláček P Estrada-Peña A Vojtíšek J Rudolf I. First record of *Hyalomma rufipes* in the Czech Republic, with a review of relevant cases in other parts of Europe. Ticks Tick Borne Dis. (2020) 11:101421. doi: 10.1016/j.ttbdis.2020.101421, 32360146

[28] Rudolf I Kejzlíková R Vojtíšek J Mendel J Peňázziová K Hubálek Z . Probable overwintering of adult *Hyalomma rufipes* in Central Europe. Ticks Tick Borne Dis. (2021) 12:101718. doi: 10.1016/j.ttbdis.2021.101718 33857747

[29] Celina SS Italiya J Tekkara AO Černý J. Crimean-Congo haemorrhagic fever virus in ticks, domestic, and wild animals. Front Vet Sci. (2025) 11:1513123. doi: 10.3389/fvets.2024.1513123, 39897158 PMC11782920

[30] Blagoveshchenskaya NM Butenko AM Vyshnivetskaya LK Zavodova TI Zarubina LV Karinskaya GA . Experimental infection of horses with Crimean hemorrhagic fever virus. Report 2. Virological and serological observations. Mater 16 Nauchn Sess Inst Polio Virusn Entsefalitov (Moscow, October 1969). (1969) 2:126–7.

[31] Milyutin V Butenko A Artyushenko A Bliznichenko A Zavodova T Zarubina LV (1969). Experimental infection of horses with Crimean hemorrhagic fever virus. Report 1: clinical observations. Mater 16 Nauchn Sess Inst Polio Virusn Entsefalitov (Moscow, October 1969), 16:145–146.

[32] Sas MA Comtet L Donnet F Mertens M Vatansever Z Tordo N . A novel double-antigen sandwich ELISA for species-independent detection of Crimean-Congo hemorrhagic fever virus antibodies. Antivir Res. (2018) 151:24–6. doi: 10.1016/j.antiviral.2018.01.006 29330092

[33] Charnley GEC Alcayna T Almuedo-Riera A Antoniou C Badolo A Bartumeus F . Strengthening resilience to emerging vector-borne diseases in Europe: lessons learnt from endemic countries. Lancet Reg Health Eur. (2025) 53:101271. doi: 10.1016/j.lanepe.2025.101271 40247854 PMC12002787

