# Supplementary material for: Equine sentinels and one health: a comprehensive serological survey of Crimean-Congo hemorrhagic fever virus in southeastern and Central Europe
Source: Front Vet Sci. 2026 Apr 24;13:1810641. doi: 10.3389/fvets.2026.1810641 (PMC13152786; doi:10.3389/fvets.2026.1810641)
Supplement: Supplementary file 1 [file Table_1.docx]

**Table S1.1.** Serological status of donkeys tested for CCHFV in Bulgaria

| **Provinces** | **Settlement** | **Latitude (°N)** | **Longitude (°E)** | **Negative** | **Positive** | **Total** |
| --- | --- | --- | --- | --- | --- | --- |
| Haskovo | Radovets | 41.931117 | 26.491973 | 21 | 4 | 25 |
|  | Studena | 41.9161917 | 26.4030288 | 5 | 7 | 12 |
| Pleven | Deventsi | 43.3212402 | 24.161508 | 20 | 1 | 21 |
| Sliven | Sadievo | 42.5301539 | 26.0771725 | 6 | 3 | 9 |
|  | Konyovo | 42.5223306 | 26.168187 | 4 | 2 | 6 |
|  | Kamenovo | 42.5422482 | 26.1016176 | 2 | 3 | 5 |
| Smolyan | Bryshlen | 41.560312 | 24.0912026 | 51 | 1 | 52 |
| Stara Zagora | FVM | 42.3983 | 25.5714 | 4 | 4 | 8 |
|  | Yasenovo | 42.6874365 | 25.2508172 | 1 | 0 | 1 |
|  | Preslaven | 42.4080681 | 25.7430894 | 2 | 0 | 2 |
|  | Gorno Botevo | 42.417167 | 25.8015305 | 9 | 3 | 12 |
|  | Han Asparuhovo | 42.4398635 | 25.8453054 | 7 | 1 | 8 |
|  | Podslon | 42.4755623 | 25.8699156 | 1 | 0 | 1 |
|  | Oryahovitsa | 42.485739 | 25.8110543 | 1 | 0 | 1 |
|  | Dalboki | 42.4812582 | 25.7705224 | 1 | 0 | 1 |
|  | Madrets | 42.1342549 | 26.0764699 | 9 | 3 | 12 |
|  | Glavan | 42.1667 | 25.7 | 17 | 2 | 19 |
| Yambol | Mamarchevo | 42.0167 | 26.7833 | 9 | 4 | 13 |
| Blagoevgrad | Pletena | 41.6167 | 24.0333 | 5 | 0 | 5 |
|  | Valkosel | 41.5833 | 24.1 | 3 | 2 | 5 |
|  | Ablanitsa | 41.5833 | 23.9333 | 3 | 5 | 8 |
|  | Blatska | 41.5333 | 23.9 | 4 | 0 | 4 |
| Sofia City | Zhelyava | 42.7833 | 23.5667 | 19 | 3 | 22 |
| Sofia Region | Madzhare | 42.2833 | 23.5833 | 11 | 2 | 13 |
| **Total** |  |  |  | **215** | **50** | **265** |

**Table S1.2.** Serological status of horses tested for CCHFV in Bulgaria

| **Provinces** | **Settlement** | **Latitude (°N)** | **Longitude (°E)** | **Negative** | **positive** | **Total** |
| --- | --- | --- | --- | --- | --- | --- |
| Blagoevgrad | Blagoevgrad | 42.013271 | 23.096880 | 1 | 0 | 1 |
|  | Pletena | 41.5333 | 24.0833 | 3 | 0 | 3 |
|  | Fargovo | 41.577947 | 24.000826 | 2 | 0 | 2 |
|  | Blatska | 41.65 | 23.95 | 1 | 0 | 1 |
|  | Satovcha | 41.618816 | 23.975906 | 2 | 0 | 2 |
|  | Ablanitsa | 41.6667 | 23.95 | 0 | 8 | 8 |
| Burgas | Ruen | 42.8 | 27.2833 | 27 | 0 | 27 |
|  | Burgas | 42.5 | 27.4667 | 1 | 0 | 1 |
|  | Marinka | 42.4 | 27.5 | 1 | 0 | 1 |
|  | Sinemorets | 42.0667 | 27.9833 | 2 | 7 | 9 |
| Haskovo | Haskovo | 41.9333 | 25.5667 | 3 | 0 | 3 |
|  | Levka | 41.6833 | 25.9333 | 50 | 13 | 63 |
| Dobrich | Dobrich | 43.5667 | 27.8333 | 10 | 1 | 11 |
| Gabrovo | Gabrovo | 42.8667 | 25.3167 | 3 | 0 | 3 |
| Montana | Valchedram | 43.7 | 23.45 | 11 | 0 | 11 |
|  | Septemvriĭtsi | 43.7333 | 23.5 | 11 | 0 | 11 |
|  | Dolni Tsibаr | 43.7333 | 23.6167 | 14 | 1 | 15 |
|  | Mokresh | 43.7333 | 23.4167 | 2 | 0 | 2 |
| Pernik | Pernik | 42.6 | 23.0333 | 5 | 0 | 5 |
| Pleven | Nikopol | 43.7 | 24.9 | 1 | 0 | 1 |
| Plovdiv | Plovdiv | 42.15 | 24.75 | 1 | 0 | 1 |
| Smolyan | Smolyan | 41.580676 | 24.706703 | 2 | 0 | 2 |
|  | Chepelare | 41.7333 | 24.6833 | 2 | 0 | 2 |
|  | Dospat | 41.647 | 24.157 | 5 | 0 | 5 |
|  | Gyovren | 41.6333 | 24.3667 | 4 | 0 | 4 |
|  | Trigrad | 41.6 | 24.3833 | 6 | 1 | 6 |
| Sofia region | Koprivshtitsa | 42.6333 | 24.35 | 41 | 1 | 42 |
|  | Ihtiman | 42.4333 | 23.8167 | 19 | 0 | 19 |
| Sofia city | Zhelyava | 42.8 | 23.6167 | 4 | 0 | 4 |
| St. Zagora | Yasenovo | 42.686359 | 25.252196 | 4 | 0 | 4 |
|  | Buzovgrad | 42.6 | 25.4 | 4 | 0 | 4 |
|  | Kazanlŭk | 42.6167 | 25.4 | 2 | 0 | 2 |
|  | Maglizh | 42.6167 | 25.5667 | 5 | 0 | 5 |
|  | FVM | 42.4177 | 25.624 | 8 | 1 | 9 |
|  | H. Dimitrovo | 42.670808 | 25.349661 | 1 | 0 | 1 |
|  | G. Cherkovishte | 42.7 | 25.6 | 2 | 0 | 2 |
| Varna | Varna | 43.2167 | 27.9167 | 9 | 1 | 10 |
| Vratsa | Lipnitsa | 43.6 | 24.05 | 11 | 0 | 11 |
| **Total** |  |  |  | **280** | **34** | **314** |

**Table S2.** Serological status of horses tested for CCHFV in Romania

| **Provinces** | **Village** | **Latitude (°N)** | **Longitude (°E)** | **Negative** | **Positive** | **Total** |
| --- | --- | --- | --- | --- | --- | --- |
| Vaslui | Mușata | 46.432389 | 28.102638 | 13 | 0 | 13 |
|  | Rânceni | 46.403546 | 28.106418 | 7 | 0 | 7 |
|  | Dumeștii vechi | 46.83212 | 27.306829 | 10 | 0 | 10 |
|  | Dumești | 46.844753 | 27.287765 | 4 | 0 | 4 |
|  | Valea mare | 46.832555 | 27.329199 | 6 | 0 | 6 |
|  | Tupilați | 46.393787 | 27.977435 | 20 | 0 | 20 |
|  | Fălciu | 46.292814 | 28.141336 | 16 | 0 | 16 |
|  | Copăceana | 46.355289 | 28.064103 | 5 | 0 | 5 |
|  | Ferești | 46.783148 | 27.697466 | 15 | 0 | 15 |
| Cluj | Gădălin | 46.824352 | 23.851682 | 1 | 0 | 1 |
|  | Jucu de sus | 46.860119 | 23.792035 | 1 | 0 | 1 |
|  | Sava | 46.881994 | 23.966813 | 12 | 0 | 12 |
|  | Mureșenii de câmpie | 46.890561 | 23.987005 | 4 | 0 | 4 |
|  | Petea | 46.865927 | 24.016263 | 3 | 0 | 3 |
|  | Pălatca | 46.843553 | 23.985771 | 34 | 0 | 34 |
|  | Câmpia turzii | 46.557851 | 23.874341 | 17 | 0 | 17 |
|  | Chinteni | 46.862108 | 23.540299 | 1 | 0 | 1 |
|  | Șoimeni | 46.958602 | 23.530300 | 8 | 0 | 8 |
|  | Băbuțiu | 46.940249 | 23.527854 | 1 | 0 | 1 |
|  | Vultureni | 46.960652 | 23.555610 | 2 | 0 | 2 |
|  | Cluj-napoca | 46.769248 | 23.652214 | 3 | 0 | 3 |
|  | Păniceni | 46.808503 | 23.185424 | 1 | 0 | 1 |
|  | Căpușu mare | 46.792561 | 23.286627 | 2 | 0 | 2 |
|  | Dăbâca | 46.971085 | 23.675960 | 3 | 0 | 3 |
|  | Pâglișa | 47.004869 | 23.640603 | 1 | 0 | 1 |
|  | Răscruci | 46.904377 | 23.769703 | 2 | 0 | 2 |
|  | Bonțida | 46.915026 | 23.816706 | 1 | 0 | 1 |
|  | Borzești | 46.539734 | 23.610820 | 1 | 0 | 1 |
| Ialomița | Gheorghe doja | 44.614970 | 27.186572 | 8 | 0 | 8 |
|  | Grivița | 44.732568 | 27.292698 | 11 | 0 | 11 |
|  | Traian | 44.764300 | 27.342941 | 1 | 0 | 1 |
|  | Smirna | 44.721931 | 27.353590 | 3 | 0 | 3 |
|  | Cocora | 44.741381 | 27.045784 | 9 | 0 | 9 |
|  | Colelia | 44.760641 | 27.007912 | 6 | 0 | 6 |
|  | Albești | 44.539803 | 27.133655 | 6 | 0 | 6 |
|  | Marsilieni | 44.539803 | 27.093658 | 9 | 0 | 9 |
|  | Axintele | 44.614183 | 26.748677 | 15 | 0 | 15 |
|  | Vlădeni | 44.607866 | 27.845614 | 10 | 0 | 10 |
|  | Săveni | 44.602957 | 27.635415 | 7 | 0 | 7 |
|  | Frățilești | 44.615824 | 27.603096 | 3 | 0 | 3 |
|  | Sudiți | 44.574571 | 27.594437 | 10 | 0 | 10 |
| Neamț | Petricani | 47.170224 | 26.470119 | 47 | 0 | 47 |
|  | Mesteacăn | 46.831894 | 27.022569 | 7 | 0 | 7 |
|  | Tabăra | 46.820898 | 27.038297 | 10 | 0 | 10 |
|  | Bălușești | 46.814179 | 26.994694 | 10 | 0 | 10 |
|  | Icușești | 26.946694 | 26.946694 | 9 | 0 | 9 |
|  | Spiridonești | 46.777389 | 26.950750 | 5 | 0 | 5 |
|  | Rocna | 46.771612 | 26.974095 | 3 | 0 | 3 |
|  | Bătrânești | 46.771055 | 26.993279 | 7 | 0 | 7 |
| Iași | Hilița | 47.006127 | 27.824933 | 2 | 0 | 2 |
|  | Gropnița | 47.358391 | 27.262550 | 9 | 0 | 9 |
|  | Hălceni | 47.443224 | 27.260985 | 3 | 0 | 3 |
|  | Șipote | 47.470776 | 27.215344 | 3 | 0 | 3 |
|  | Mitoc | 47.462486 | 27.237145 | 3 | 0 | 3 |
|  | Miroslava | 47.149192 | 27.522972 | 3 | 0 | 3 |
|  | Stornești | 47.117924 | 27.172214 | 4 | 0 | 4 |
|  | Vocotești | 47.081358 | 27.468051 | 2 | 0 | 2 |
|  | Proselnici | 47.108799 | 27.483211 | 2 | 0 | 2 |
|  | Cornești | 47.105667 | 27.513037 | 4 | 0 | 4 |
|  | Podu hagiului | 46.861246 | 28.081354 | 6 | 0 | 6 |
|  | Cornești | 47.105608 | 27.512694 | 3 | 0 | 3 |
|  | Băiceni | 47.301464 | 26.917598 | 2 | 0 | 2 |
|  | Stroiești | 47.323900 | 26.890947 | 2 | 0 | 2 |
|  | Todirești | 47.325374 | 26.836615 | 3 | 0 | 3 |
|  | Hărmăneștii vechi | 47.272062 | 26.806983 | 1 | 0 | 1 |
|  | Boldești | 47.281760 | 26.825652 | 2 | 0 | 2 |
|  | Gorban | 46.881046 | 28.079584 | 3 | 0 | 3 |
|  | Scopoșeni | 47.133176 | 27.410416 | 8 | 0 | 8 |
|  | Hărmăneștii noi | 47.252954 | 26.797990 | 1 | 0 | 1 |
|  | Butea | 47.069767 | 26.935708 | 2 | 0 | 2 |
|  | Gura bohotin | 46.909508 | 28.053717 | 7 | 0 | 7 |
|  | Comarna | 47.067763 | 27.776763 | 7 | 0 | 7 |
|  | A.i.cuza | 47.132819 | 26.857665 | 3 | 0 | 3 |
|  | Kogălniceni | 47.158117 | 26.839159 | 2 | 0 | 2 |
|  | Tăutești | 47.232148 | 27.462032 | 4 | 0 | 4 |
|  | Șcheia | 47.115947 | 26.882064 | 3 | 0 | 3 |
|  | Volintirești | 47.171502 | 26.829010 | 2 | 0 | 2 |
|  | Ciortești | 46.913359 | 27.845169 | 2 | 0 | 2 |
| Brăila | Însurăței | 44.913997 | 27.605831 | 98 | 0 | 98 |
| Teleorman | Banov | 44.445423 | 25.336103 | 4 | 0 | 4 |
|  | Preajba | 44.432707 | 25.358934 | 4 | 0 | 4 |
|  | Poeni | 44.403676 | 25.341038 | 16 | 0 | 16 |
|  | Cătunu | 44.424554 | 25.320482 | 4 | 0 | 4 |
|  | Țăvârlău | 44.453603 | 25.335373 | 1 | 0 | 1 |
|  | Ciuperceni | 43.756370 | 24.929394 | 10 | 0 | 10 |
|  | Cosmești | 44.315237 | 25.378365 | 9 | 0 | 9 |
|  | Dobreni | 44.421413 | 25.125775 | 6 | 0 | 6 |
|  | Tătărăștii de sus | 44.407390 | 25.122353 | 4 | 0 | 4 |
|  | Pietroșani | 43.714818 | 25.637154 | 25 | 0 | 25 |
|  | Bujoru | 43.710586 | 25.559563 | 15 | 0 | 15 |
| Bihor | Șimian | 47.487036 | 22.086590 | 3 | 0 | 3 |
|  | Batăr | 46.710115 | 21.802904 | 2 | 0 | 2 |
|  | Tăut | 46.719329 | 21.834290 | 2 | 0 | 2 |
|  | Arpășel | 46.741859 | 21.718339 | 1 | 0 | 1 |
|  | Vaida | 47.252417 | 21.994816 | 10 | 0 | 10 |
|  | Sântimreu | 47.246927 | 22.043296 | 4 | 0 | 4 |
|  | Sălard | 47.220461 | 22.034025 | 2 | 0 | 2 |
|  | Hodoș | 47.223221 | 21.983384 | 4 | 0 | 4 |
|  | Cheț | 47.420520 | 22.337844 | 5 | 0 | 5 |
|  | Sebiș | 46.618229 | 22.433202 | 5 | 0 | 5 |
|  | Petrani | 46.690987 | 22.265692 | 3 | 0 | 3 |
|  | Poiana | 47.292815 | 22.348599 | 1 | 0 | 1 |
|  | Criștioru de jos | 46.417882 | 22.534267 | 4 | 0 | 4 |
|  | Dumbrăvița | 46.793426 | 22.153791 | 1 | 0 | 1 |
|  | Codru | 46.640594 | 22.139358 | 3 | 0 | 3 |
|  | Feneriș | 46.714384 | 22.281517 | 2 | 0 | 2 |
|  | Ursad | 46.677680 | 22.086381 | 6 | 0 | 6 |
|  | Brusturi | 47.158810 | 22.237358 | 4 | 0 | 4 |
|  | Păulești | 47.173449 | 22.216887 | 1 | 0 | 1 |
|  | Sălbociu | 47.073336 | 22.153544 | 2 | 0 | 2 |
|  | Țețchea | 47.045401 | 22.318038 | 1 | 0 | 1 |
|  | Hotar | 47.020026 | 22.281646 | 2 | 0 | 2 |
|  | Tămașda | 46.643292 | 21.553330 | 3 | 0 | 3 |
|  | Avram iancu | 46.669137 | 21.525478 | 2 | 0 | 2 |
|  | Fughiu | 47.058777 | 22.046663 | 4 | 0 | 4 |
|  | Alparea | 47.027779 | 22.055772 | 1 | 0 | 1 |
|  | Aleșd | 47.058747 | 22.398333 | 4 | 0 | 4 |
|  | Tinăud | 47.052200 | 22.434768 | 1 | 0 | 1 |
|  | Tarcea | 47.458986 | 22.171273 | 3 | 0 | 3 |
|  | Galoșpetreu | 47.483420 | 22.216286 | 2 | 0 | 2 |
|  | Curtuișeni | 47.548566 | 22.201383 | 5 | 0 | 5 |
|  | Tinca | 46.775696 | 21.931800 | 5 | 0 | 5 |
| Ilfov | Berceni | 44.315483 | 26.186448 | 90 | 0 | 90 |
| Giurgiu | Singureni | 44.232836 | 25.945120 | 103 | 0 | 103 |
|  | Hotarele | 44.176537 | 26.366586 | 7 | 0 | 7 |
|  | Herăști | 44.216384 | 26.357660 | 11 | 0 | 11 |
| Mureș | Poienița | 46.555713 | 24.667482 | 3 | 0 | 3 |
|  | Corunca | 46.519901 | 24.611128 | 3 | 0 | 3 |
|  | Agrișteu | 46.392395 | 24.683924 | 8 | 0 | 8 |
|  | Vețca | 46.351800 | 24.789512 | 1 | 0 | 1 |
|  | Jacodu | 46.329716 | 24.830365 | 4 | 0 | 4 |
|  | Bordoșiu | 46.396013 | 24.784641 | 3 | 0 | 3 |
|  | Fântânele | 46.421631 | 24.766745 | 1 | 0 | 1 |
|  | Sovata | 46.599312 | 25.075799 | 10 | 0 | 10 |
|  | Trei sate | 46.476782 | 24.898881 | 10 | 0 | 10 |
|  | Abud | 46.524868 | 24.913607 | 1 | 0 | 1 |
|  | Solocma | 46.477944 | 24.995130 | 6 | 0 | 6 |
|  | Chibed | 46.532611 | 24.961323 | 13 | 0 | 13 |
|  | Sărățeni | 46.561994 | 25.011609 | 4 | 0 | 4 |
| Brașov | Vama buzăului | 45.586254 | 25.987654 | 1 | 0 | 1 |
|  | Râșnov | 45.575128 | 25.449975 | 3 | 0 | 3 |
|  | Recea | 45.732002 | 24.939849 | 4 | 0 | 4 |
|  | Lisa | 45.727453 | 24.849080 | 4 | 0 | 4 |
|  | Predeal | 45.506257 | 25.576857 | 6 | 0 | 6 |
|  | Săcele | 45.617248 | 25.692664 | 25 | 0 | 25 |
|  | Sânpetru | 45.710198 | 25.635384 | 27 | 0 | 27 |
|  | Bod | 45.763430 | 25.640471 | 1 | 0 | 1 |
|  | Hărman | 45.711709 | 25.687327 | 25 | 0 | 25 |
| Olt | Strejești (positive) | 44.527624 | 24.268584 | 5 | 1 | 6 |
|  | Giuvărăști | 43.793152 | 24.685871 | 10 | 0 | 10 |
| Sibiu | Sebeșu de jos | 45.648936 | 24.334201 | 10 | 0 | 10 |
|  | Sebeș de sus (positive) | 45.652716 | 24.355144 | 3 | 1 | 4 |
|  | Daia | 45.801265 | 24.285771 | 2 | 0 | 2 |
|  | Vurpăr | 45.893580 | 24.340896 | 1 | 0 | 1 |
|  | Boița | 45.633635 | 24.258134 | 16 | 0 | 16 |
|  | Agnita (positive) | 45.975862 | 24.626897 | 19 | 1 | 20 |
|  | Dealu frumos | 45.987061 | 24.698660 | 21 | 0 | 21 |
|  | Miercurea sibiului | 45.889891 | 23.789230 | 3 | 0 | 3 |
|  | Dobârca | 45.852017 | 23.774805 | 15 | 0 | 15 |
|  | Apoldu de sus | 45.851340 | 23.825354 | 1 | 0 | 1 |
|  | Racovița | 45.680608 | 24.343170 | 7 | 0 | 7 |
| Tulcea | Alecsandri | 44.795327 | 28.502869 | 7 | 0 | 7 |
|  | Beidaud | 44.714409 | 28.571006 | 9 | 1 | 10 |
|  | Calfa | 44.824072 | 28.350602 | 13 | 0 | 13 |
|  | Cerna | 45.076596 | 28.303210 | 25 | 9 | 34 |
|  | Ceamurlia de jos | 44.747108 | 28.725509 | 2 | 1 | 3 |
|  | Fagarasu nou | 44.857124 | 28.285475 | 12 | 0 | 12 |
|  | G-ral praporgescu | 45.011741 | 28.375830 | 1 | 3 | 4 |
|  | Luminita | 44.892886 | 28.322665 | 42 | 1 | 43 |
|  | Magurele | 44.900343 | 28.236494 | 17 | 0 | 17 |
|  | Mircea voda | 45.037504 | 28.359446 | 9 | 4 | 13 |
|  | Neatarnarea | 44.744091 | 28.507867 | 5 | 0 | 5 |
|  | Peceneaga | 45.003144 | 28.153395 | 29 | 1 | 30 |
|  | Topolog | 44.878071 | 28.371737 | 63 | 0 | 63 |
|  | Traian | 45.026319 | 28.232060 | 8 | 0 | 8 |
| **Total** |  |  |  | **1511** | **23** | **1534** |

**Table S3.** Serological status of horses tested for CCHFV across the Czech Republic

| **Village** | **Latitude (°N)** | **Longitude (°E)** | **No. of samples tested** |
| --- | --- | --- | --- |
| Benešov | 49.7817 | 14.6862 | 6 |
| Beroun | 49.963 | 14.072 | 17 |
| Blansko | 49.3635 | 16.6448 | 3 |
| Břeclav | 48.7583 | 16.882 | 9 |
| Brno-město | 49.1952 | 16.608 | 6 |
| Brno-venkov | 49.2768 | 16.5754 | 3 |
| České Budějovice | 48.9747 | 14.4743 | 5 |
| Chrudim | 49.9512 | 15.7955 | 1 |
| Domažlice | 49.4404 | 12.9316 | 72 |
| Havlíčkův Brod | 49.607 | 15.58 | 43 |
| Jihlava | 49.396 | 15.5912 | 53 |
| Jindřichův Hradec | 49.1446 | 15.003 | 38 |
| Klatovy | 49.3954 | 13.2956 | 23 |
| Kutná Hora | 49.9481 | 15.2682 | 10 |
| Litoměřice | 50.5333 | 14.131 | 32 |
| Pelhřimov | 49.4317 | 15.223 | 62 |
| Písek | 49.3088 | 14.1475 | 4 |
| Plzeň-jih | 49.5825 | 13.378 | 2 |
| Plzeň-sever | 49.9041 | 13.3775 | 13 |
| Praha-město | 50.0755 | 14.4378 | 4 |
| Praha-východ | 50.136 | 14.651 | 35 |
| Příbram | 49.6891 | 14.0104 | 7 |
| Strakonice | 49.2615 | 13.9022 | 9 |
| Svitavy | 49.755 | 16.468 | 32 |
| Tábor | 49.4144 | 14.6578 | 22 |
| Tachov | 49.798 | 12.634 | 7 |
| Třebíč | 49.215 | 15.881 | 26 |
| Ústí nad Orlicí | 49.973 | 16.393 | 8 |
| Žďár nad Sázavou | 49.5626 | 15.939 | 11 |
| Znojmo | 48.8555 | 16.0488 | 13 |
| **Total** |  |  | **576** |
